# Inter-laminar microcircuits across neocortex: repair and augmentation

**DOI:** 10.3389/fnsys.2013.00080

**Published:** 2013-11-19

**Authors:** Ioan Opris

**Affiliations:** Department of Physiology and Pharmacology, Wake Forest University School of MedicineWinston-Salem, NC, USA

**Keywords:** cortical minicolumn, cortical layer, cortical module, microcircuit, neocortex, repair, brain machine interface, prosthetics

## Introduction

Repair and brain augmentation approaches, such as brain-machine interfaces, neural stimulation and other neural prostheses, have experienced a rapid development during the last decade (Nicolelis et al., 2003; Lebedev and Nicolelis, 2006). Still, only few of these methods target the fine microcircuitry of the brain (Jones and Rakic, 2010; Opris et al., 2012a). Here, it is highlighted the potential employing of inter-laminar recording and microstimulation of cortical microcircuits to build neural prostheses for repair and augmentation of cognitive function. In the future, such microcircuit-based prostheses will provide efficient therapies for patients with neurological and psychiatric disorders. Moreover, it is implied that neural enhancement approaches can be applied to inter-laminar microcircuits across the entire cortex.

## Cortical microcircuits

**Figure 1:**
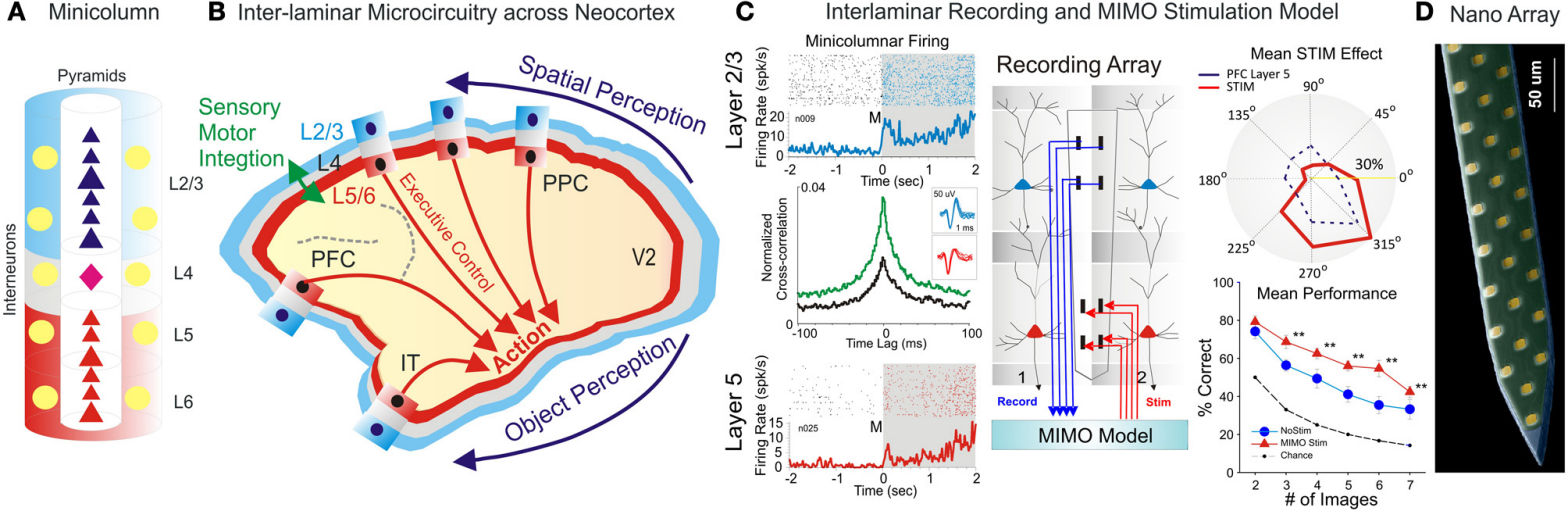
**Inter-Laminar Microcircuits across the Neocortex. (A)** Cortical minicolumn with pyramidal cells labeled in dark blue for supra-granular layers and red for infra-granular layers. Stellate cells in layer 4 are colored in pink. The “curtain of inhibition” is depicted by interneurons, colored in yellow. **(B)** Primate brain showing the cortical mantle split in cortical layers and minicolumns. Minicolumn across neocortex work cooperatively to translate perception into complex action. **(C)** Interlaminar recording of pyramidal cells and MIMO stimulation model. Rasters and peri-event histograms in blue and red depict the activity of supra-and infra-granular layers. Cross-correlation show that inter-laminar firing increased following the presentation of targets compared to pre-target epoch. Recording array with the MIMO model for recording in layer 2/3 and stimulation in layer 5. Stimulation effect compare the population tuning for MIMO stim (red) vs. layer 5 prefrontal cortical activity (dark blue dotted line). Overall MIMO stimulation effect (red) is significantly greater than no-stim and the chance level (with permission from Opris et al., 2012a,b, 2013). **(D)** Nanoarray for recording neural activity in cortical layers and minicolumns (with permission from Alivisatos et al., 2013). ^**^p < 0.001, ANOVA.

As proposed by Mountcastle, the primate neocortical circuitry has a modular architecture that subserves a multitude of sensory (visual, auditory, touch), motor, cognitive (attention, memory, decision) and emotional functions (Mountcastle, 1957, 1997; Opris and Bruce, 2005; Shepherd and Grillner, 2010). These modules are composed of elementary building blocks formed by vertical arrangements of cortical neurons, called minicolumns (Szentágothai and Arbib, 1975; Mountcastle, 1997). Within minicolumns, cortical neurons are aggregated into six horizontal layers (or laminae): three supra-granular layers (L1-L3), a granular layer (L4) and two infra-granular layers (L5/L6) (Figure 1A). The granular layer receives sensory input from thalamus (Constantinople and Bruno, 2013). The supra-granular layers consist of small pyramidal neurons that form a complex network of intra-cortical connections, particularly the connections to the infra-granular layers of larger pyramidal neurons that generate most of the output from cerebral cortex to other parts of the brain (Buxhoeveden and Casanova, 2002). According to this three stratum functional module, infra-granular layers execute the associative computations elaborated in supra-granular layers (Buxhoeveden and Casanova, 2002; Casanova et al., 2011).

Here, the focus is on inter-laminar cortical microcircuits formed by interconnected pyramidal neurons from the supra-granular and infra-granular layers (Thomson and Bannister, 2003; Opris et al., 2011, 2012a,b, 2013). These microcircuits receive input from neurons in layer L4, which project to L2/3, or through direct thalamic projections to the supragranular layers in the higher-order cortical areas. Neurons in L2/3 then project top-down to L5, where they target specific types of pyramidal cells and inhibitory interneurons. Some L5 neurons project back to L2/3 neurons, forming an inter-laminar loop (Weiler et al., 2008) or back to L4, targeting mostly interneurons (Thomson and Bannister, 2003). The outputs from cortical microcircuits, cortico-striatal projections arise mostly from L5, whereas cortico-thalamic projections arise from L6.

Cortical microcircuits are strikingly similar across the neocortex (hence the term “canonical microcircuits”). It has been suggested that such repeatability in the microcircuit pattern plays a key role in reducing the errors of encoding (Bastos et al., 2012). Some characteristics of microcolumns are specific to particular cortical areas. For example, the thickness of L4 is different across areas (DeFelipe et al., 2012). It is most prominent in sensory areas and the thinnest in the motor cortex. There are also area-specific differences in the topographic connectivity of microcircuits with their cortical and subcortical projection areas (Das and Gilbert, 1995; Kritzer and Goldman-Rakic, 1995; Opris et al., 2013).

### Inter-area connectivity

Cortical microcircuits are connected into a macro-network by cortico-cortical connections, which link areas within the same hemisphere, as well as between hemispheres (Van Essen et al., 1982). This super network subserves the “perception-to-action” cycle—a group of processes that handle environmental stimuli and convert them into actions (Romo et al., 2002; Fuster and Bressler, 2012). Microcircuits within the same hemisphere are interconnected (from low level sensory to high level associative processes) through horizontal connections in lamina 2/3, spanning over many cortical areas (Das and Gilbert, 1995; Kritzer and Goldman-Rakic, 1995; Fuster and Bressler, 2012).

Inter-area connectivity of cortical microcircuits preserves spatial topography suggesting a column-to-column match from one area to another (e.g., Figure 1B schematics of V1 projections to prefrontal area 46 through the dorsal visual stream; Goldman-Rakic, 1996). Additionally, the topography is preserved within minicolumns owing to the inter-laminar projections (Opris et al., 2013). Interhemispheric connectivity is formed by neural interconnections of lamina 3b (Jones et al., 1979; Van Essen et al., 1982).

## Microcircuits and cognition

Recent research conducted in non-human primates indicates that a variety of sensory, motor and executive functions emerge from the interactions between frontal, parietal, temporal and occipital cortical microcircuits (Atencio and Schreiner, 2010; Buffalo et al., 2011; Takeuchi et al., 2011; Hansen et al., 2012; Opris et al., 2012a,b, 2013; Hirabayashi et al., 2013a,b; Mahan and Georgopoulos, 2013). Moreover, several augmentation approaches based on microcircuits have been implemented. These advances have been possible owing to the development of new multi-electrode arrays (MEA) fitted for recordings from neural elements of cortical columns (Moxon et al., 2004). Thus, MEAs with linear or bi-linear geometry have been successfully employed for simultaneous recordings from supra- and infragranular cortical laminae in adjacent minicolumns, resulting in unprecedented insights into the function of cortical microcircuits (Mo et al., 2011; Opris et al., 2011, 2012a,b, 2013).

A number of recent publications suggest that cortical microcircuits perform elementary computations while cognitive functions are sub-served by a broader network comprising multiple cortical areas (Fuster and Bressler, 2012). For example, elementary computations related to executive control are performed by microcircuits in the prefrontal cortex (Opris et al., 2012a,b), whereas microcircuits of the temporal cortex maintain long term memory (Takeuchi et al., 2011; Hirabayashi et al., 2013a). Prefrontal microcircuits are in a unique and privileged position at the top of sensory-to-motor hierarchy network because they coordinate a multitude of stimuli, perceptions, biases and actions related to such functions as attention, decision making, and working memory. As such, prefrontal microcicuits integrate and synthetize signals over a broad spectrum of perceptual stimuli and various modalities. This integration is performed in supra-granular layers, whereas the output of the infra-granular layers provides selection-related signals, which are sent back to the infra-granular layers and the other areas comprising the network. As a matter of fact, signals can reverberate within inter-laminar loops. Thus, cortical microcircuits for long term memory in entorhinal cortex and hippocampal formation employ such reverberating signals (Takeuchi et al., 2011) to integrate relevant information over time (Fuster, 2001).

Our group at Wake Forest University in collaboration with Dr. Berger's team at USC and Dr. Gerhard's group at University of Kentucky, examined the executive function of prefrontal microcircuits (Opris et al., 2012a,b, 2013). We trained rhesus monkeys to select a target (spatial or object) for hand movement, after a memory delay, while the neural activity in prefrontal microcircuits was recorded (Figure 1C). Our electrode arrays were specifically designed to record from neurons located in both supra- & infra-granular layers of adjacent minicolumns. We analyzed correlated firing in neurons from the supra- and infra-granular layers. Interestingly, the extent of correlated firing was linked to the accuracy of monkey performance. Correlated firing between cell pairs within single minicolumns was higher during correct selections and reduced in error trials (Opris et al., 2012a). Thus, we discovered that animals make errors when their prefrontal cortical microcircuits do not function properly when handle task relevant information. Additionally, we discovered that during the presentation of the target and during the executive selection of the correct target, assemblies of cell firing in prefrontal layers exhibited similar tuning to target locations on behavioral trials in which this information was important. These studies provided a direct demonstration of real-time inter-laminar processing of information in prefrontal microcircuits during decision-making (Opris and Bruce, 2005; Opris et al., 2012a).

## Cognitive enhancement approaches based on microcircuits

Recent studies have demonstrated that cognitive enhancement can be achieved by microstimulation of specific elements of cortical microcircuits (Opris et al., 2001, 2013; Hampson et al., 2012). These enhancement methods employed a multi-input/multi-output (MIMO) Volterra kernel-based non-linear dynamic model, which was applied to the spatiotemporal patterns of neuronal firing recorded in prefrontal cortical layers L2/3 and L5 to convert the firing of neurons in layer 2/3 into microstimulation patterns applied to layer 5 (Berger et al., 2011; Hampson et al., 2012). MIMO model is based on the principle of multiplexing, where a high rate signal is split into several low rate signals, which are then sent to multiple recipients via multiple channels. Using multiple channels of information transfer MIMO model provides a more reliable communication (Figure 1C, right panel).

To perform cognitive augmentation, inter-laminar recordings are analyzed via a non-linear MIMO model, whose output is then converted into patterns of microstimulation (Berger et al., 2011). In these studies, MIMO models used a precise *topographically matched stimulation* by extracting the patterns of firing that relate to the successful behavioral performance. This allowed the substitution of task-related laminar L5 neuron firing patterns with electrical stimulation in the same recording regions during columnar transmission from lamina L2/3 at the time of target selection. Such stimulation improved normal task performance, but more importantly, recovered performance after being impaired by a pharmacological disruption of decision making (Hampson et al., 2012). Moreover, the fact that stimulation-induced spatial preference (in percent correct performance) on spatial trials that was similar to neural tuning indicated that inter-laminar prefrontal microcircuits played causal roles to the executive function (Opris et al., 2005, 2013). These findings provided the first successful demonstration of a microcircuit-based neuroprosthesis designed specifically to restore or repair disrupted cognitive function.

## Neurological diseases and microcircuits

Disruption of inter-laminar microcircuits within cortical minicolums is a signature of a broad spectrum of neurological and psychiatric disorders, such as autism (Casanova, 2013), schizophrenia (Di Rosa et al., 2009), Alzheimer's disease (Chance et al., 2011) drug addiction (Opris et al., 2012a) and other disorders. The use of both invasive MIMO stimulation (Hampson et al., 2012) and non-invasive transcranial magnrtic stimulation (TMS; Sokhadze et al., 2012) are valuable potential options to repair or treat such dysfunctions. The multitude of deficits in a cortical microcircuit involve the micro-anatomic disconnections between layers or within minicolumns (autism, schizophrenia, Alzheimer), the intra- and inter-laminar neuromodulation (drug addiction, aging), the lack or excess of inhibition (ADHD, depression), etc.

Microcircuit-based neuroprostheses, such as MIMO based memory implants (Berger et al., 2011), and decision chips (Hampson et al., 2012) hold the promise to provide treatment for neurological conditions that result from compromised microcircuits. Targeting cortical microcircuitry may be key to the development of next-generation enhancement methods and medical treatments.

## Future directions for microcircuit-based approaches

An emerging approach with broad implications for basic and clinical neuroscience is based on optogenetic stimulation (Gradinaru et al., 2007; Tye and Deisseroth, 2012). Recent developments in optogenetics based on optical manipulation of activity in neural circuits with light-sensitive rhodopsins, such as the *Chlamydomonas* channelrhodopsin-2 (ChR2) are now capable to stimulate the inter-laminar microcircuits at millisecond-scale, with cell type-specific effects of optical perturbations in non-human primates (Diester et al., 2011; Han, 2012), opening up new possibilities for repair and augmentation.

Recent developments in nanotechnological tools and in the design and synthesis of nano-materials have generated optical, electrical, and chemical methods that can readily be adapted for use in neuroscience. Nanotechnology was instrumental to nanofabricated planar electrode array (Figure 1D) for high-density neuronal voltage recording (Du et al., 2011; Suyatin et al., 2013). Leveraging micro- and nanofabrication technology raises the prospect for creating vastly greater numbers of electrodes and smaller, less invasive implantable devices. A promising category for brain microcircuits is the planar electrode array (Viventi et al., 2011; Alivisatos et al., 2013), which is patterned on a crystalline, ceramic, or polymer support structure (Figure 1D). The recording of neuronal activity with three-dimensional (3D) microelectrode arrays (Zorzos et al., 2012) represents a major advance in brain activity mapping techniques, by providing a tool to probe how intra and inter-laminar/regional neural circuits cooperate to process information. Building prosthetic minicolumns as basic modules to repair the damaged cortical tissue will become a valuable approach in the cognitive neuroprosthetics.

To trace the flow of neural signals in the cortical microcircuits across neocortex, or in the large scale brain networks, analytical tools based on dynamic Bayesian networks and Granger causality are available (Granger, 1969; Smith et al., 2006). These methods allow to identify putative causal interactions and population codes within the neural circuits involved in perception and behavior (Yu et al., 2004; Beck et al., 2008).

Microcircuit-based augmentation could be implemented in several cortical areas, where different functions could be enhanced. Thus, the prefrontal cortical microcircuits involved in attention, working memory, executive decisions and conflict monitoring may be augmented for autism (Casanova et al., 2010), schizophrenia (Chance et al., 2011), drug addiction (Opris et al., 2012a), Alzheimer's or attention deficit disorders.

In conclusion, a better understanding of the function of inter-laminar microcircuits across the neocortex is needed for the development of treatments for neurological disorders, as well as for the development of methods of brain augmentation.
